# Distinct fatty acid signatures in infrapatellar fat pad and synovial fluid of patients with osteoarthritis versus rheumatoid arthritis

**DOI:** 10.1186/s13075-019-1914-y

**Published:** 2019-05-22

**Authors:** Anne-Mari Mustonen, Reijo Käkelä, Petri Lehenkari, Johanna Huhtakangas, Sanna Turunen, Antti Joukainen, Tommi Kääriäinen, Tommi Paakkonen, Heikki Kröger, Petteri Nieminen

**Affiliations:** 1 Institute of Biomedicine, School of Medicine, Faculty of Health Sciences, University of Eastern Finland, P.O. Box 1627, FI-70211 Kuopio, Finland; 2 Department of Environmental and Biological Sciences, Faculty of Science and Forestry, University of Eastern Finland, P.O. Box 111, FI-80101 Joensuu, Finland; 3 Molecular and Integrative Biosciences Research Programme, Faculty of Biological and Environmental Sciences, University of Helsinki, P.O. Box 65, FI-00014 Helsinki, Finland; 4 Helsinki University Lipidomics Unit (HiLIPID), Helsinki Institute for Life Science (HiLIFE), University of Helsinki, P.O. Box 65, FI-00014 Helsinki, Finland; 5 Cancer and Translational Medicine Research Unit, Faculty of Medicine, University of Oulu, P.O. Box 5000, FI-90014 Oulu, Finland; 6 Department of Surgery and Medical Research Center, Oulu University Hospital (OYS), P.O. Box 21, FI-90029 Oulu, Finland; 7 Rheumatology Unit and Medical Research Center, Oulu University Hospital (OYS), P.O. Box 21, FI-90029 Oulu, Finland; 8 Department of Orthopaedics, Traumatology and Hand Surgery, Kuopio University Hospital (KYS), P.O. Box 100, FI-70029 Kuopio, Finland

**Keywords:** Fatty acid, Infrapatellar fat pad, n-3 polyunsaturated fatty acids, n-6 polyunsaturated fatty acids, Osteoarthritis, Rheumatoid arthritis, Synovial fluid

## Abstract

**Background:**

Infrapatellar fat pad (IFP) has recently emerged as a potential source of inflammation in knee arthropathies. It has been proposed to be one source of adipocytokines, fatty acids (FA), and FA-derived lipid mediators that could contribute to the pathophysiological processes in the knee joint. Alterations in synovial fluid (SF) lipid composition have been linked to both osteoarthritis (OA) and rheumatoid arthritis (RA). The aim of the present study was to compare the FA signatures in the IFP and SF of RA and OA patients.

**Methods:**

Pairs of IFP and SF samples were collected from the same knees of RA (*n* = 10) and OA patients (*n* = 10) undergoing total joint replacement surgery. Control SF samples (*n* = 6) were harvested during diagnostic or therapeutic arthroscopic knee surgery unrelated to RA or OA. The FA composition in the total lipids of IFP and SF was determined by gas chromatography with flame ionization and mass spectrometric detection.

**Results:**

Arthropathies resulted in a significant reduction in the SF proportions of n-6 polyunsaturated FA (PUFA), more pronouncedly in OA than in RA. OA was also characterized with reduced percentages of 22:6n-3 and lower product/precursor ratios of n-3 PUFA. The proportions of total monounsaturated FA increased in both RA and OA SF. Regarding IFP, RA patients had lower proportions of 20:4n-6, total n-6 PUFA, and 22:6n-3, as well as lower product/precursor ratios of n-3 PUFA compared to OA patients. The average chain length of SF FA decreased in both diagnoses and the double bond index in OA.

**Conclusions:**

The observed complex alterations in the FA signatures could have both contributed to but also limited the inflammatory processes and cartilage destruction in the RA and OA knees.

## Background

The potential inflammatory role of the infrapatellar fat pad (IFP) has recently become a topic of interest for osteoarthritis (OA) [1] and rheumatoid arthritis (RA) research [2]. IFP is an intracapsular but extrasynovial organ that does not directly interact with the articular cartilage [3]. It has, however, been proposed to be a source of fatty acids (FA), FA-derived lipid mediators (LM), and adipocytokines that could contribute to the pathophysiological processes in knee arthropathies [1, 2, 4]. IFP-conditioned medium is able to stimulate OA fibroblast-like synoviocytes to produce prostaglandin E_2_ (PGE_2_), cytokines, and cartilage-degrading enzymes [5], which suggests a role as an anatomical site in the complex process of knee joint inflammation. RA synovial tissues typically have a more inflammatory phenotype with more infiltrating immune cells and a higher expression of cytokines than OA joints [6]. The IFP of RA patients is also infiltrated by a higher number of immune cells, while the types and levels of secreted adipocytokines are similar to OA [2].

Articular synovial fluid (SF) is the synovial membrane-produced ultrafiltrate of blood plasma that contains lubricating compounds, such as hyaluronan and phospholipids (PL). The concentrations of cholesterol, lipoproteins, and apolipoproteins increase in RA SF [7]. In addition, the levels of various glycero-PL and sphingolipids are elevated in RA and OA SF [8, 9]. FA and their derivatives have been proposed to play roles in the pathophysiology of joint diseases [10]. Changes in the desaturation and chain length of FA could affect the anti-friction and lubricating properties of surface-active PL that cover the articular cartilage [11, 12]. Local FA concentrations could also contribute to inflammatory processes and cartilage degradation [10]. Generally, n-6 polyunsaturated FA (PUFA), which are overrepresented in the Western diet [13, 14], are precursors to pro-inflammatory LM, while n-3 PUFA are converted to less inflammatory or resolving ones [10, 15]. In fact, n-3 PUFA, especially 20:5n-3 (eicosapentaenoic acid), have anti-inflammatory and anti-destructive effects on cartilage [16, 17]. In agreement with this, dietary fish oil supplements containing high proportions of 20:5n-3 and 22:6n-3 (docosahexaenoic acid) can reduce the tender joint count and morning stiffness in RA patients [18]. Increased consumption of long-chain n-3 PUFA may also have beneficial effects on pain alleviation and function of OA joints, although the evidence is less convincing [19].

The major dietary n-6 PUFA, 18:2n-6 (linoleic acid), when first converted to 20:4n-6 (arachidonic acid), can have pro-inflammatory effects on cartilage via increased PGE_2_ production [20]. OA joints have been documented to accumulate n-6 PUFA, especially 20:4n-6, the immediate precursor of PGE_2_ [21, 22]. The major monounsaturated FA (MUFA), 18:1n-9 (oleic acid), inhibits cartilage destruction, while the role for the most common saturated FA (SFA), 16:0 (palmitic acid), remains controversial [20, 23]. According to Lu et al. [24], a high dietary intake of SFA can be associated with the progression of knee OA, while dietary PUFA and MUFA have potentially protective effects. PGE_2_ levels can be elevated in SF/plasma of RA and OA patients [10]. However, pro-resolving LM can be also detected in both RA and OA joints, where they could potentially contribute to the resolution pathways [25–27].

Previous studies focusing on the detailed assessment of FA signatures in RA or OA joints are scarce. To assess the contribution of IFP to knee arthropathies, our aim was to examine the proportions of a comprehensive array of FA in the IFP and SF of RA and OA patients. To the best of our knowledge, this is the first time the FA profiles of IFP and SF sample pairs from the same knees are compared between RA and OA. It was hypothesized that RA would be characterized with a more pro-inflammatory FA signature compared to OA in both IFP and SF.

## Methods

### Subjects, ethics, and sampling

Patients with knee joint disorders (seropositive RA: men *n* = 3, women *n* = 7; primary OA: men *n* = 2, women *n* = 8; Table 1) were recruited at the Oulu University Hospital with the permission of the Ethical Committee of the Hospital (decision #29/2011, amendment 2/24/2014) in compliance with the Helsinki Declaration. As the selection criteria, we only sampled patients who had reached the age of majority (18 years) and were undergoing knee surgery for pre-existing medical indications (Table 2). Prior to the surgery, all patients signed consent forms to donate their IFP and SF samples. General data were recorded as follows: sex, age, body mass, height, body mass index (BMI), operation, operative diagnosis, and medication. No data that would enable the identification of the patients were registered.

**Table 1:** Demographic characteristics of the sampled knee surgery patients (mean ± SEM)

Group	Control	RA	OA	*p* group	*p* sex	*p* interaction
Sex	2 M, 4 F	3 M, 7 F	2 M, 8 F	0.869		
Age (years)	34 ± 4	72 ± 3*	66 ± 3*	< 1 × 10^−15^	0.935	0.005
Body mass (kg)	76.3 ± 7.19	67.2 ± 5.97	82.6 ± 4.53	0.622	0.666	0.064
BMI (kg/m^2^)	26.0 ± 2.26	24.9 ± 1.50	31.2 ± 1.76	0.326	0.062	0.021

Sex ratios were tested with the Fisherʼs exact test

*RA* rheumatoid arthritis, *OA* osteoarthritis, *M* male, *F* female, *BMI* body mass index

*Significant difference from control (generalized linear model)

**Table 2:** Clinical characteristics and diagnoses of the sampled knee surgery patients

ID	Group	Gender	Operation	Operative diagnosis
01	Control	M	Arthroscopy, MPFL reconstruction	M22.0
02	Control	F	Arthroscopy, MPFL reconstruction	M22.0
03	Control	F	Arthroscopy, ACL reconstruction	M23.5, M23.2
04	Control	F	Diagnostic arthroscopy	M23.5
05	Control	F	Arthroscopy, debridement	M25.5
06	Control	M	Arthroscopy, partial resection of med. men.	M23.2
07	RA	F	Total knee replacement	M17.5 (secondary)
08	RA	F	Total knee replacement	M17.5 (secondary)
09	RA	F	Total knee replacement	M17.5 (secondary)
10	RA	F	Total knee replacement	M17.5 (secondary)
11	RA	M	Total knee replacement	M17.5 (secondary)
12	RA	F	Total knee replacement	M17.4 (other secondary)
13	RA	F	Total knee replacement	M17.4 (other secondary)
14	RA	M	Total knee replacement	M17.5 (secondary)
15	RA	M	Total knee replacement	M17.5 (secondary)
16	RA	F	Total knee replacement	M17.4 (other secondary)
17	OA	M	Total knee replacement	M17.1 (primary)
18	OA	M	Total knee replacement	M17.1 (primary)
19	OA	F	Total knee replacement	M17.1 (primary)
20	OA	F	Total knee replacement	M17.1 (primary)
21	OA	F	Total knee replacement	M17.1 (primary)
22	OA	F	Total knee replacement	M17.1 (primary)
23	OA	F	Total knee replacement	M17.1 (primary)
24	OA	F	Total knee replacement	M17.1 (primary)
25	OA	F	Total knee replacement	M17.1 (primary)
26	OA	F	Total knee replacement	M17.1 (primary)

*M* male, *F* female, *RA* rheumatoid arthritis, *OA* osteoarthritis, *MPFL* medial patellofemoral ligament, *ACL* anterior cruciate ligament, *med. men.* medial meniscus, *M22.0* recurrent dislocation of patella, *M23.5* chronic instability of the knee, *M23.2* derangement of meniscus due to old tear or injury, *M25.5* pain in joint, *M17.5* other secondary gonarthrosis, *M17.4* other secondary gonarthrosis, bilateral, *M17.1* other primary gonarthrosis

SF samples were collected with sterile needles and syringes during joint replacement surgery performed at the Oulu University Hospital in 2011–2017 and stored at − 70 °C. IFP samples were harvested from the same patients during the removal of periarticular tissues according to the demands of the joint replacement procedure. All samples were obtained during surgery for pre-existing indications, and they represented tissues that would have been removed during surgery regardless of the study. The total duration of the surgery increased only by a minimal amount of time due to sampling. For comparison, control SF samples (men *n* = 2, women *n* = 4) were collected during arthroscopic knee surgery performed as diagnostic arthroscopy or due to trauma without underlying RA/OA at the Kuopio University Hospital in 2014 with the permission of the Ethical Committee of the Hospital (decision #79//2013) in compliance with the Helsinki Declaration. The samples were stored at − 80 °C. The selection criteria for control patients and the collection of general data were similar to patients with RA and OA.

### FA analyses

Subsamples of IFP and SF were transmethylated in methanolic H_2_SO_4_ under nitrogen atmosphere [28], and the formed FA methyl esters (FAME) were extracted with hexane and analyzed by a Shimadzu GC-2010 Plus gas chromatograph (Shimadzu, Kyoto, Japan) employing flame ionization detector (FID). The FAME structures were confirmed by using electron impact mass spectra recorded by a Shimadzu GCMS-QP2010 Ultra with a mass selective detector. The resulting chromatographic peaks from FID were manually integrated with the GCsolution software (*v*2.41.00) by Shimadzu. The results represent the FA composition (mol-%) of IFP and SF total lipids, and when calculating indices, as defined below, the mol-% values of the FA were always used. The n-6 and n-3 PUFA product/precursor ratios were calculated as follows: (20:3n-6 + 20:4n-6)/18:2n-6 and (20:5n-3 + 22:6n-3)/18:3n-3. The double bond index (DBI) was calculated by using the formula: [(1 × (Σ MUFA)) + (2 × (Σ dienoic FA)) + (3 × (Σ trienoic FA)) + (4 × (Σ tetraenoic FA)) + (5 × (Σ pentaenoic FA)) + (6 × (Σ hexaenoic FA))]/100. The total average chain length (TACL) was calculated as follows: [(14 × (Σ 14C FA)) + (15 × (Σ 15C FA)) + (16 × (Σ 16C FA)) + (17 × (Σ 17C FA)) + (18 × (Σ 18C FA)) + (19 × (Σ 19C FA)) + (20 × (Σ 20C FA)) + (22 × (Σ 22C FA)) + (24 × (Σ 24C FA))]/100. The ∆9-desaturation index was calculated with the formula: [(14:1n-5 + 16:1n-9 + 16:1n-7 + 16:1n-5 + 17:1n-8 + 18:1n-9 + 18:1n-7 + 18:1n-5 + 20:1n-11 + 20:1n-9 + 20:1n-7 + 22:1n-11 + 22:1n-9 + 22:1n-7 + 24:1n-9)/(14:0 + 16:0 + 17:0 + 18:0 + 20:0 + 22:0 + 24:0)], which included all the MUFA irrespective of whether their production also required chain-elongation or chain-shortening steps after the desaturation of the SFA precursor. The ∆6-desaturation index was calculated as 18:3n-6/18:2n-6 (since 18:4n-3 did not accumulate in the studied tissues, 18:4n-3/18:3n-3 ratio was not used) and the ∆5-desaturation index as 20:4n*-*6/20:3n-6 and 20:5n-3/20:4n-3.

### Statistical analyses

Comparisons between the study groups were performed with the generalized linear model executed with the normal probability distribution (IBM SPSS *v*25 software, IBM, Armonk, NY, USA). The examined parameter was the dependent variable and the study group the model factor. Relative differences in the FA proportions between IFP and SF were tested with the independent samples *t* test. Sex ratios in the study groups were tested with Fisherʼs exact test. Correlations were calculated with the Spearman correlation coefficient (*r*_s_). The *p* value < 0.05 was considered statistically significant. The results are presented as the mean ± standard error of the mean (SEM). To carry out a general assessment of the FA profiles, we also performed the discriminant analysis by classifying FA data by discriminant functions to determine how the samples in the study groups differed from one another, which variables separated them most clearly, and how well the analysis was able to classify the samples into their respective study groups: control, RA, or OA.

## Results

General demographic data of the patients are presented in Table 1. The RA and OA patients were significantly older than those in the control group. The body masses or BMI were not affected by the diagnoses. Neither did sex have an effect on the general characteristics of the patients, but there was a significant group × sex interaction regarding age and BMI.

**Fig. 1:**
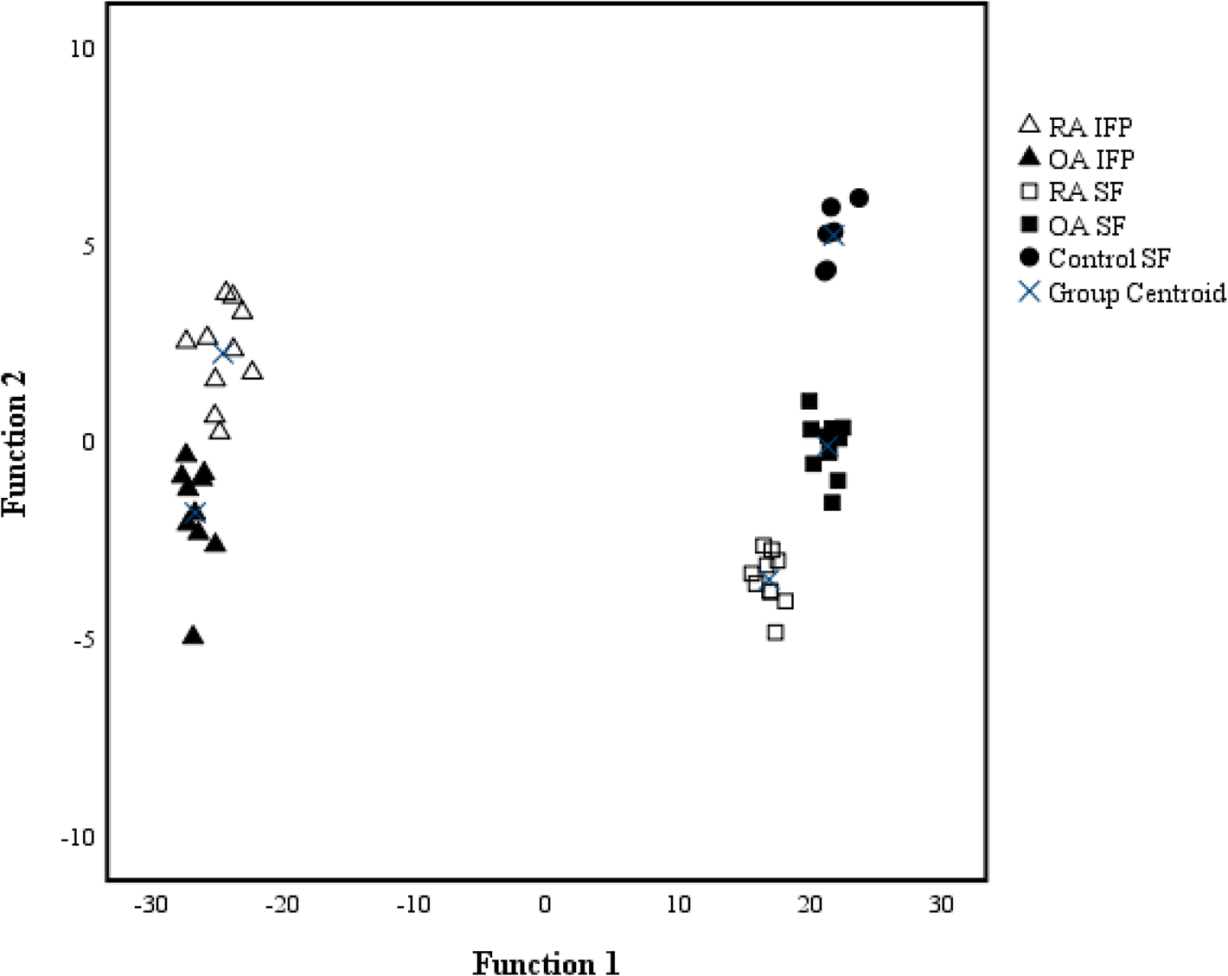
Discriminant analysis depicting the classification of fatty acid signatures in the studied tissues based on discriminant functions 1 and 2. With these first two functions, 98.4% of the variance was explained. IFP infrapatellar fat pad, SF synovial fluid, OA osteoarthritis, RA rheumatoid arthritis. Note that the scaling is different in the *x*- and *y*-axes

In the discriminant analysis, the five study groups were clearly separated from each other based on the FA signatures (Fig. 1). The FA having the largest separation power included 18:2n-6, 22:4n-6, 18:3n-3, 22:6n-3, 18:1n-9, and 20:4n-6. The analysis classified 100% of the samples correctly into their respective study groups. The general differences in the FA profiles between IFP and SF can be examined in Table 3.

**Table 3:** Percentages (mol-%) of individual fatty acids (FA), sums of different FA classes, ratios, and indices in synovial fluid (SF) and infrapatellar fat pad (IFP; mean ± SEM)

	Control SF	RA SF	OA SF	RA IFP	OA IFP	*p*
14:0	3.310 ± 0.272	3.778 ± 0.154	3.801 ± 0.217	3.733 ± 0.195	3.174 ± 0.150^‡, #^	0.033
14:1n-5	0.224 ± 0.035	0.342 ± 0.045	0.330 ± 0.026*	0.617 ± 0.073^#^	0.495 ± 0.065^#^	0.000001
15:0	0.230 ± 0.013	0.264 ± 0.020	0.285 ± 0.014*	0.344 ± 0.024^#^	0.313 ± 0.020	0.001
DMA 16:0	0.096 ± 0.018	0.091 ± 0.008	0.092 ± 0.007	0.016 ± 0.005^#^	0.037 ± 0.012^#^	3.1 × 10^−14^
16:0	28.222 ± 0.772	28.823 ± 0.417	29.301 ± 0.727	23.216 ± 1.205^#^	24.604 ± 0.644^#^	5.5 × 10^−11^
16:1n-9	0.400 ± 0.032	0.416 ± 0.015	0.374 ± 0.023	0.626 ± 0.039^#^	0.741 ± 0.050^#^	< 10^−20^
16:1n-7	2.011 ± 0.130	2.725 ± 0.351	2.867 ± 0.414	7.670 ± 0.821^#^	6.732 ± 0.789^#^	3.3 × 10^−16^
16:1n-5	0.217 ± 0.029	0.327 ± 0.031*	0.232 ± 0.025^†^	0.170 ± 0.019^#^	0.195 ± 0.034	0.000222
17:0*i*	0.106 ± 0.005	0.112 ± 0.018	0.119 ± 0.015	0.064 ± 0.005^#^	0.063 ± 0.010^#^	0.000254
17:0*ai*	0.251 ± 0.028	0.330 ± 0.026*	0.304 ± 0.023	0.213 ± 0.011^#^	0.215 ± 0.023^#^	0.000022
17:0	0.587 ± 0.053	0.531 ± 0.029	0.589 ± 0.050	0.211 ± 0.033^#^	0.263 ± 0.040^#^	< 10^−20^
17:1n-8	0.157 ± 0.025	0.196 ± 0.014	0.188 ± 0.023	0.297 ± 0.014^#^	0.299 ± 0.022^#^	6.0 × 10^−10^
DMA 18:0	0.347 ± 0.032	0.374 ± 0.024	0.316 ± 0.036	0.041 ± 0.018^#^	0.078 ± 0.022^#^	< 10^−20^
DMA 18:1n-9	0.301 ± 0.047	0.353 ± 0.046	0.329 ± 0.041	0.031 ± 0.016^#^	0.081 ± 0.034^#^	1.9 × 10^−15^
DMA 18:1n-7	0.029 ± 0.004	0.059 ± 0.007*	0.065 ± 0.009*	0.010 ± 0.005^#^	0.025 ± 0.009^#^	1.4 × 10^−9^
18:0	13.640 ± 0.843	14.077 ± 0.729	15.041 ± 1.159	3.784 ± 0.673^#^	5.137 ± 0.848^#^	< 10^−20^
18:1n-9	22.996 ± 1.009	25.107 ± 0.764	26.424 ± 1.225*	44.581 ± 1.198^#^	40.945 ± 1.738^#^	< 10^−20^
18:1n-7	2.700 ± 0.138	3.006 ± 0.049	3.010 ± 0.108	2.989 ± 0.167	3.262 ± 0.193	0.124
18:1n-5	0.668 ± 0.056	0.834 ± 0.079	0.718 ± 0.073	0.181 ± 0.056^#^	0.201 ± 0.052^#^	< 10^−20^
18:2n-7	0.278 ± 0.036	0.229 ± 0.030	0.243 ± 0.022	0.212 ± 0.023	0.180 ± 0.016	0.087
18:2n-6	14.460 ± 2.395	10.205 ± 0.983*	8.291 ± 1.056*	7.198 ± 0.231^#^	8.068 ± 0.473	0.00001
18:3n-6	0.066 ± 0.023	0.112 ± 0.046	0.123 ± 0.062	0.031 ± 0.011	0.047 ± 0.008	0.276
19:1n-8	0.077 ± 0.020	0.073 ± 0.010	0.098 ± 0.018	0.077 ± 0.009	0.063 ± 0.007	0.323
18:3n-3	0.592 ± 0.063	0.659 ± 0.045	0.610 ± 0.031	0.846 ± 0.041^#^	0.773 ± 0.074^#^	0.001
18:2*c*9*t*11	0.093 ± 0.031	0.082 ± 0.011	0.105 ± 0.043	0.179 ± 0.064^#^	0.292 ± 0.097^#^	0.049
20:0	0.294 ± 0.034	0.244 ± 0.009	0.266 ± 0.029	0.086 ± 0.012^#^	0.087 ± 0.020^#^	< 10^−20^
20:1n-11	0.156 ± 0.042	0.120 ± 0.016	0.212 ± 0.057	0.191 ± 0.022	0.193 ± 0.038	0.358
20:1n-9	0.336 ± 0.162	0.207 ± 0.026	0.229 ± 0.042	0.569 ± 0.028^#^	0.411 ± 0.031^‡, #^	6.3 × 10^−7^
20:1n-7	0.130 ± 0.048	0.107 ± 0.012	0.166 ± 0.086	0.087 ± 0.007	0.092 ± 0.007	0.644
20:2n-9	0.176 ± 0.107	0.101 ± 0.016	0.073 ± 0.005	0.041 ± 0.012^#^	0.057 ± 0.009	0.048
20:2n-6	0.229 ± 0.035	0.193 ± 0.023	0.190 ± 0.043	0.116 ± 0.038	0.281 ± 0.077	0.128
20:3n-9	0.039 ± 0.007	0.042 ± 0.007	0.044 ± 0.011	0.014 ± 0.004^#^	0.019 ± 0.004^#^	0.002
20:3n-6	0.743 ± 0.137	0.676 ± 0.056	0.586 ± 0.070	0.139 ± 0.021^#^	0.171 ± 0.027^#^	< 10^−20^
20:4n-6	3.159 ± 0.594	2.470 ± 0.227	1.790 ± 0.269*^, †^	0.337 ± 0.097^#^	0.742 ± 0.176^‡, #^	< 10^−20^
20:4n-3	0.619 ± 0.057	0.741 ± 0.075	0.769 ± 0.130	0.122 ± 0.043^#^	0.192 ± 0.052^#^	7.6 × 10^−14^
20:5n-3	0.419 ± 0.087	0.355 ± 0.067	0.289 ± 0.048	0.069 ± 0.011^#^	0.092 ± 0.014^#^	2.9 × 10^−9^
22:0	0.205 ± 0.022	0.268 ± 0.057	0.311 ± 0.054	0.083 ± 0.043^#^	0.177 ± 0.068	0.016
22:1n-11	0.040 ± 0.006	0.046 ± 0.006	0.045 ± 0.005	0.025 ± 0.007^#^	0.024 ± 0.005^#^	0.002
22:1n-9	0.129 ± 0.008	0.131 ± 0.016	0.121 ± 0.012	0.057 ± 0.013^#^	0.063 ± 0.014^#^	8.9 × 10^−7^
22:1n-7	0.028 ± 0.005	0.025 ± 0.003	0.035 ± 0.006	0.010 ± 0.004^#^	0.010 ± 0.003^#^	0.000002
22:4n-6	0.041 ± 0.010	0.033 ± 0.004	0.125 ± 0.064	0.105 ± 0.018^#^	0.227 ± 0.038^‡, #^	0.001
22:5n-3	0.078 ± 0.014	0.128 ± 0.028	0.172 ± 0.042	0.350 ± 0.138	0.366 ± 0.054^#^	0.014
24:0	0.388 ± 0.020	0.331 ± 0.018*	0.282 ± 0.025*	0.044 ± 0.024^#^	0.068 ± 0.018^#^	< 10^−20^
22:6n-3	0.626 ± 0.097	0.556 ± 0.065	0.327 ± 0.059*^, †^	0.191 ± 0.036^#^	0.396 ± 0.040^‡^	8.6 × 10^−8^
24:1n-9	0.107 ± 0.017	0.122 ± 0.015	0.108 ± 0.018	0.029 ± 0.008^#^	0.048 ± 0.008^#^	1.2 × 10^−8^
SFA	47.232 ± 1.936	48.758 ± 1.287	50.301 ± 2.024	31.778 ± 1.781^#^	34.102 ± 1.471^#^	< 10^−20^
MUFA	30.377 ± 1.306	33.783 ± 1.100*	35.159 ± 1.638*	58.174 ± 1.891^#^	53.774 ± 1.825^#^	< 10^−20^
PUFA	21.618 ± 3.251	16.581 ± 1.251	13.739 ± 1.263*	9.950 ± 0.488^#^	11.902 ± 0.574^‡^	6.8 × 10^−9^
n-6 PUFA	18.699 ± 3.102	13.689 ± 1.201	11.106 ± 1.267*	7.927 ± 0.260^#^	9.536 ± 0.466^‡^	2.6 × 10^−8^
n-3 PUFA	2.334 ± 0.182	2.439 ± 0.135	2.167 ± 0.178	1.578 ± 0.225^#^	1.819 ± 0.114	0.000484
n-9 PUFA	0.215 ± 0.106	0.143 ± 0.014	0.117 ± 0.013	0.055 ± 0.016^#^	0.075 ± 0.010^#^	0.009
DMA	0.773 ± 0.078	0.877 ± 0.069	0.801 ± 0.079	0.098 ± 0.042^#^	0.221 ± 0.076^#^	< 10^−20^
UFA/SFA	1.118 ± 0.082	1.046 ± 0.056	1.004 ± 0.089	2.247 ± 0.207^#^	1.971 ± 0.117^#^	< 10^−20^
n-3/n-6 PUFA	0.148 ± 0.029	0.192 ± 0.021	0.220 ± 0.035	0.197 ± 0.022	0.191 ± 0.009	0.393
Prod/prec n-6 PUFA	0.267 ± 0.012	0.313 ± 0.014	0.300 ± 0.036	0.068 ± 0.017^#^	0.122 ± 0.032^#^	< 10^−20^
Prod/prec n-3 PUFA	1.834 ± 0.355	1.408 ± 0.160	1.023 ± 0.135*	0.320 ± 0.062^#^	0.692 ± 0.099^‡, #^	5.5 × 10^−12^
TACL	17.26 ± 0.06	17.16 ± 0.02*	17.14 ± 0.03*	17.18 ± 0.03	17.20 ± 0.03	0.044
DBI	0.87 ± 0.07	0.79 ± 0.03	0.72 ± 0.04*	0.82 ± 0.02	0.84 ± 0.01^#^	0.010
Δ9-DI	0.65 ± 0.01	0.71 ± 0.04	0.73 ± 0.07	1.96 ± 0.19^#^	1.64 ± 0.11^#^	< 10^−20^
Δ6-DI n-6 PUFA	0.005 ± < 0.01	0.02 ± 0.01	0.02 ± 0.02	0.004 ± < 0.01	0.006 ± < 0.01	0.323
Δ5-DI n-6 PUFA	4.28 ± 0.60	3.68 ± 0.23	3.07 ± 0.32	2.22 ± 0.25	5.38 ± 1.67	0.058
Δ5-DI n-3 PUFA	0.75 ± 0.20	0.53 ± 0.09	0.42 ± 0.08	0.76 ± 0.11	0.66 ± 0.12	0.134

*RA* rheumatoid arthritis, *OA* osteoarthritis, *DMA* dimethyl acetal, *i.e.* plasmalogen alkenyl chain derivative, *i* iso, *ai* anteiso, *c* cis, *t* trans, *SFA* saturated fatty acid, *MUFA* monounsaturated fatty acid, *PUFA* polyunsaturated fatty acid, *UFA* unsaturated fatty acid (MUFA + PUFA), *prod* product, *prec* precursor, *TACL* total average chain length, *DBI* double bond index, *Δ9-DI* Δ9-desaturation index, *Δ6-DI* Δ6-desaturation index, *Δ5-DI* Δ5-desaturation index

*Significant difference from the control SF

^†^Significant difference between RA and OA SF

^‡^Significant difference between RA and OA IFP

^#^Significant difference between IFP and SF within a diagnosis (generalized linear model)

According to the generalized linear model, RA SF had higher proportions of 16:1n-5, 17:0*ai*, DMA 18:1n-7 (dimethyl acetal), and total MUFA compared to control SF, while 18:2n-6, 24:0, and TACL were lower (Table 3). Regarding OA SF, the proportions of 14:1n-5, 15:0, DMA 18:1n-7, 18:1n-9, and total MUFA were elevated and those of 18:2n-6, 20:4n-6, 24:0, 22:6n-3, total PUFA, and total n-6 PUFA reduced, and the product/precursor ratios of n-3 PUFA, TACL, and DBI decreased compared to control SF. RA SF had higher percentages of 16:1n-5, 20:4n-6, and 22:6n-3 than OA SF. Regarding IFP, RA lipids had higher proportions of 14:0 and 20:1n-9 but lower percentages of 20:4n-6, 22:4n-6, 22:6n-3, total PUFA, and n-6 PUFA, and decreased product/precursor ratios of n-3 PUFA.

The n-3/n-6 PUFA ratios of SF correlated positively with the n-3/n-6 PUFA ratios of IFP in RA patients (*r*_s_ = 0.903, *p* < 0.0004) but not in OA patients (*r*_s_ = 0.200, *p* = 0.580). Similarly, the n-3 PUFA sums correlated positively between SF and IFP in RA patients (*r*_s_ = 0.709, *p* = 0.022) but not in OA patients (*r*_s_ = 0.321, *p* = 0.365), and the 18:1n-9 proportions between SF and IFP in OA patients (*r*_s_ = 0.648, *p* = 0.043) but not in RA patients (*r*_s_ = − 0.321, *p* = 0.365). In addition, the BMI correlated inversely with the SF proportions of 22:6n-3 in RA patients (*r*_s_ = − 0.723, *p* = 0.018) but not in OA patients (*r*_s_ = − 0.406, *p* = 0.244). There were no significant correlations between the BMI and n-3 or n-6 PUFA sums, nor the n-3/n-6 PUFA ratios in SF or IFP (*r*_s_ = − 0.479–0.200, *p* = 0.162–0.947) except for the n-6 PUFA sums in the IFP of RA group (*r*_s_ = − 0.729, *p* = 0.017). In the generalized linear model with BMI as a covariate, the effects of the BMI on the SF and IFP n-3 PUFA sums, n-6 PUFA sums, and n-3/n-6 PUFA ratios were nonsignificant (*p* = 0.266–0.566).

## Discussion

The significance of age, sex, and obesity in joint disorders is well-documented, which was also confirmed in the discriminant analysis of the present study. However, the novelty of our results is in the FA part of the study and in the potential dialogue between the FA profiles and inflammatory phenomena in the two major joint diseases, RA and OA. The main findings of the present study were as follows: (i) n-6 PUFA reduced in proportion in RA SF and especially in OA SF compared to control knees, (ii) 22:6n-3 and product/precursor ratios of n-3 PUFA decreased in OA SF, (iii) TACL and DBI reduced especially in OA SF, and (iv) total n-6 PUFA, 20:4n-6, 22:6n-3, and product/precursor ratios of n-3 PUFA were lower in RA IFP than in OA IFP.

It was initially hypothesized that the RA knee would exhibit a more pro-inflammatory FA signature compared to the OA knee. However, we observed a loss of n-6 PUFA in the SF of both RA and OA patients, as well as lower 20:4n-6 and total n-6 PUFA proportions in RA IFP compared to OA IFP. There is a growing consensus for a detrimental role of n-6 PUFA and their derivatives in joint health. It has been established that n-6 PUFA, especially 20:4n-6, can accumulate in OA joints [21, 22] and that they increase cyclooxygenase-2 protein levels [29] and PGE_2_ production in chondrocytes [20]. In plasma PL, 20:4n-6 levels were positively associated with the degree of knee synovitis [30], and low dietary 20:4n-6 intake reduced inflammation in RA patients [31]. In addition, the secretion of 20:4n-6 was higher from the IFP of OA patients compared to post-mortem donors without OA [4]. However, the situation is more complex, as n-6 PUFA also have potential roles in the pathways of resolution of inflammation [32]. Previously, 18:3n-6 (gamma-linolenic acid) was noted to improve signs and symptoms of RA disease activity [33], and in a recent study, 18:2n-6 levels in erythrocytes were inversely associated with the risk of RA [34]. According to Van de Vyver et al. [35], 20:4n-6 concentrations decreased in the SF free FA of patients with end-stage knee OA, supporting the present results.

To the best of our knowledge, the observed reduction in the proportions of n-6 PUFA has not been previously documented for RA SF or IFP, but some similarities have been reported for the circulating levels of n-6 PUFA in RA patients [36–38]. It is tempting to hypothesize that the decreased relative proportions of n-6 PUFA in SF could reduce inflammation and, in this way, provide a potentially protective mechanism in knee arthropathies. Alternatively, the reduced SF proportions of 18:2n-6 and 20:4n-6 could have been due to their intensified conversion to short-lived LM. This view is supported by the SF activity of phospholipase A_2_ [39], an enzyme that cleaves PUFA from PL for LM synthesis. In addition, based on the few liquid chromatography–tandem mass spectrometry studies conducted, SF contains several pro- and anti-inflammatory LM originating from n-6 and n-3 PUFA [25, 40].

In addition to the loss of n-6 PUFA, we documented reduced 22:6n-3 proportions in OA SF, also reflected in the decreased n-3 PUFA product/precursor ratios. Previous data have shown that n-3 PUFA can have protective effects on joint tissues, for instance, by reducing the production of cyclooxygenase-2, inflammatory cytokines, and cartilage-degrading enzymes [16, 17, 29] and, for these reasons, the relative decrease of 22:6n-3 in the OA SF samples was expected. Earlier, the mobilization of 22:6n-3 from IFP was higher in OA patients than in post-mortem donors without OA [4]. As 22:6n-3 is a precursor of pro-resolving LM, it could contribute to the resolution pathways in diseased joints [25–27]. The n-3 PUFA levels in the SF of RA patients of the present study were not different from controls, and their 22:6n-3 percentages were somewhat higher than in OA SF. This is opposite to the findings of Navarro et al. [37], who documented decreased n-3 PUFA levels in the SF of RA patients compared to those with OA. These data agree with the literature in which a higher dietary intake of long-chain n-3 PUFA has been associated with a lower risk of developing RA [41] and in which dietary fish oil supplements have reduced tender joint count and stiffness of RA joints [18]. In contrast to our SF results, the lower 22:6n-3 proportions in RA IFP compared to OA IFP would be in line with earlier literature.

A high n-6/n-3 PUFA ratio in the diet and in the body is associated with obesity and several other pathological conditions [13, 14]. Our research group observed an increase in the IFP n-6/n-3 PUFA ratio due to experimentally induced early OA in a rabbit model [42] and, in human plasma, a high n-6/n-3 PUFA ratio was associated with greater pain and functional limitations of the OA knee [43], suggesting implications in joint diseases. However, there was no relationship between SF n-3/n-6 PUFA ratios and OA severity or synovitis in an obese mouse model of OA [44]. In the present study, the n-3/n-6 PUFA ratios in SF and IFP were unaffected by RA or OA, which could mean that both types of PUFA are used for LM production, also supported by the previous liquid chromatography–tandem mass spectrometry profiling studies of SF LM [25, 27, 40]. We did not find increased product/precursor ratios of n-6 PUFA, different from previous literature on serum PL [36, 38]. Neither were there statistically significant differences in the ∆6- or ∆5-desaturation indices according to the diagnoses. These results are in line with the study by de Pablo et al. [34], who found no significant association between these indicators of PUFA metabolism and the risk of RA.

The present results also revealed an increase in the SF proportions of total MUFA in both RA and OA. Previously, Navarro et al. [37] documented elevated percentages of long-chain MUFA in RA SF, possibly in association with increased sphingomyelin levels. In the present study, 18:1n-9 showed increased percentages in OA SF and, previously, it was identified as a critical metabolite for discriminating between early- and late-stage OA with increased levels in SF during disease progression [45]. The possible role for MUFA in joint diseases is not clear, but 18:1n-9 has exerted anti-destructive effects on chondrocytes and cartilage in vitro [20]. In this way, the elevated MUFA proportions could counteract pathologic processes in RA and OA.

A high dietary intake of SFA was previously connected to the disease progression of knee OA [24] and the major SFA, 16:0, induced inflammation, articular cartilage breakdown, and chondrocyte apoptosis in vitro [23]. However, 16:0 was also documented to inhibit cartilage destruction [20]. A recent study using an obese mouse model of OA reported negative association of SF SFA with OA severity [44]. We did not find significant changes in the proportions of major SFA between RA and OA SF or IFP, while Navarro et al. [37] observed increased 16:0 and total SFA levels in the SF of RA compared to OA patients. To sum up the present results, the major differences between the control knees and the RA and OA joints were an increase in MUFA and a simultaneous decrease in n-6 PUFA. It has been previously proposed that IFP could inhibit catabolic processes in the end-stage OA [46]. Together, these findings suggest that in addition to destructive cascades in the diseased joint, protective biochemical phenomena may also arise, when a share of n-6 PUFA is replaced with MUFA, perhaps following intense consumption of n-6 PUFA precursors for LM production. These observations have potential translational applications, as altering the FA profile could help alleviate the pathological processes in both RA and OA.

Even though PL levels in SF have been documented to be elevated in RA and OA [8, 9], articular surfaces have been proposed to become deficient in surface-active PL in OA [47]. Changes in the desaturation and length of FA chains could influence the anti-friction and lubricating properties of these PL covering the articular cartilage [11, 12]. Kosinska et al. [12] suggested that there could be an increase in the PL species with longer FA chains in RA and OA, which could hypothetically reduce friction and protect articular surfaces. We found decreased TACL in both RA and OA SF and reduced DBI in OA SF. Provided that PL with longer-chain and more unsaturated FA would reduce the coefficient of friction in joints [48], the decreased TACL and DBI could be examples of harmful FA alterations in RA and OA, in contrast to the observed decrease in n-6 PUFA and the increase in MUFA.

**Fig. 2:**
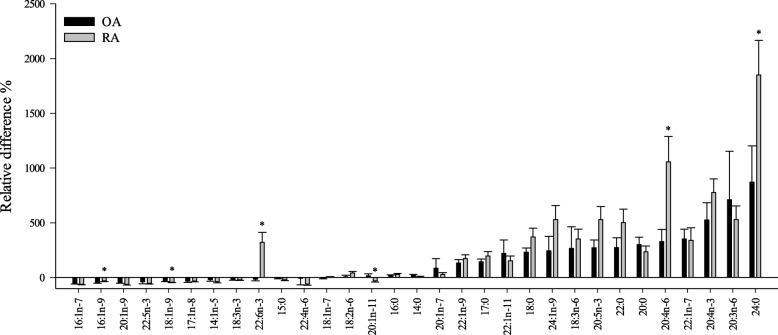
The relative differences (%) in the proportions of selected fatty acids (FA) between tissues. IFP infrapatellar fat pad, SF synovial fluid, OA osteoarthritis, RA rheumatoid arthritis. The bars with negative values indicate that a FA had higher percentages in IFP than in SF, and the bars with positive values indicate higher percentages in SF than in IFP [(mol-% in SF–mol-% in IFP)/mol-% in IFP; mean + SEM]. Asterisk denotes significant difference between OA and RA (*t* test, *p* < 0.05)

It has been proposed that most SF FA would derive from plasma [37], but IFP has also been suggested to be one source of FA and LM affecting the knee joint [4, 5, 49]. IFP has been assumed to induce both protective and disease-aggravating activities in the OA knee [49]. The interrelations between the IFP and SF FA profiles were assessed here by the simultaneous analysis of these tissues from the same knees, and the relative differences in the proportions of FA between IFP and SF are represented in Fig. 2. The proportions of several long-chain SFA and MUFA were higher in SF, and their source could possibly be plasma sphingomyelin known to harbor these FA [50]. We only found a few significant correlations in the FA proportions between IFP and SF. This could indicate that the release of FA from IFP may not be as prominent as could be assumed or that it is mostly the FA-derived LM that are released from IFP to SF via the synovial membrane. SF lipids may also derive directly from tissue destruction in diseased joints causing the IFP and SF FA profiles to diverge.

When investigating the reasons behind the tissue- and diagnosis-specific differences in the FA composition, the relative importance of the changes due to systemic PUFA distribution in the body vs. locally different PUFA metabolism is difficult to address. However, no tissues of any wild type mammals are able to convert n-6 PUFA to n-3 PUFA or vice versa, a trait only possible through introduced genes from invertebrates [51]. Still, these structurally different PUFA families affect each other’s metabolism by competing for the same enzymatic conversions, especially for the further ∆6- and ∆5-desaturations of PUFA precursors that already have the n-3 or n-6 double bond in place [52]. However, this competition that can be found in the structural modifications of PUFA does not seem to occur between n-3 and n-6 PUFA in the cycles of incorporation and hydrolysis that remodel the lipid acyl chains [53]. In addition, different phospholipases have preferred substrates, and the hydrolysis rates depend on the lipid molecule head group and acyl chains [54]. Thus, specific PUFA may be hydrolyzed and consumed at different rates when cyclooxygenase, lipoxygenase, and cytochrome P450 monooxygenase enzymes convert n-6 and n-3 PUFA to various LM [55, 56].

Some potential limitations regarding the present study should be acknowledged. The control group for SF was relatively small and, in addition, the IFP samples unfortunately lacked a control group. This was due to ethical reasons, as IFP is not routinely removed during arthroscopic surgery unlike in total joint replacement. Furthermore, the control SF samples do not represent healthy but RA- and OA-free joints, and knee trauma can influence the SF lipid composition [57]. In addition, the control patients were significantly younger than the RA and OA groups, which is quite unavoidable considering the demographics of degenerative joint diseases vs. knee trauma. The effects of age on body FA profiles cannot be excluded [58]. While this variable may be a confounding factor regarding the study population, it would be logistically difficult to obtain an age-standardized sample population regarding knee diseases. By the time of the knee replacement surgery, the patients had a long disease history that had been treated with systemic and local medications, and we cannot exclude the possibility that some of these could have influenced the FA profiles, especially regarding the usually more intensively medicated RA patients.

## Conclusions

The FA signatures in IFP and SF were clearly different between the studied diagnoses. These changes could contribute to disease progression by influencing inflammatory processes, cartilage destruction, and friction in the joint. The FA manifestations of RA and OA could not be regarded as strictly pro-inflammatory or ameliorating but likely reflected both inflammation and protective biochemical phenomena. The major changes from the control knees to the RA and OA joints were an increase in MUFA and a simultaneous decrease in n-6 PUFA. There is accumulating evidence for a protective role of n-3 PUFA in joint health, but the potential roles of the n-6 PUFA loss and the increase in MUFA should be investigated in future studies on RA and OA.

## Abbreviations

16:0: Palmitic acid
18:1n-9: Oleic acid
18:2n-6: Linoleic acid
18:3n-6: Gamma-linolenic acid
20:4n-6: Arachidonic acid
20:5n-3: Eicosapentaenoic acid
22:6n-3: Docosahexaenoic acid
BMI: Body mass index
DBI: Double bond index
DMA: Dimethyl acetal
FA: Fatty acid
FAME: Fatty acid methyl ester
FID: Flame ionization detector
IFP: Infrapatellar fat pad
MUFA: Monounsaturated fatty acid
OA: Osteoarthritis
PGE_2_: Prostaglandin E_2_
PL: Phospholipid
PUFA: Polyunsaturated fatty acid
SFA: Saturated fatty acid
TACL: Total average chain length
TAG: Triacylglycerol
UFA: Unsaturated fatty acid
